# Preparation and Properties of SBR Composites Containing Graphene Nanoplatelets Modified with Pyridinium Derivative

**DOI:** 10.3390/ma13235407

**Published:** 2020-11-27

**Authors:** Magdalena Gaca, Cyril Vaulot, Magdalena Maciejewska, Magdalena Lipińska

**Affiliations:** 1 Department of Chemistry, Institute of Polymer and Dye Technology, Lodz University of Technology, 12/16 Stefanowskiego Str., 90-924 Lodz, Poland; magdalena.maciejewska@p.lodz.pl (M.M.); magdalena.lipinska@p.lodz.pl (M.L.); 2 Institut de Science des Matériaux de Mulhouse (IS2M), Université de Haute-Alsace, CNRS UMR 7361, F-68100 Mulhouse, France; cyril.vaulot@uha.fr; 3 Université de Strasbourg, F-67081 Strasbourg, France

**Keywords:** ionic liquids, composites preparation, SBR, graphene nanoplatelets, fillers modification, composites properties

## Abstract

The goal of this work was to study the effect of graphene nanoplatelets (GnPs) modified with ionic liquid (IL) on properties of styrene–butadiene rubber (SBR) composites. GnPs were decorated with IL or were modified in bulk directly during rubber mix preparation. The ionic liquid used was 1-butyl-4-methylpyridinium tetrafluoroborate (BMPFB). The textural properties were studied to confirm surface modification of GnPs with BMPFB. In these investigations, the impact of the concentration of GnPs and the effect of the method of GnPs’ modification with IL on elastomers properties are described. Some thermal measurements (e.g., differential scanning calorimetry and thermogravimetry) were conducted to characterize the thermal stability or the vulcanization process of the investigated samples. Complementary experimental techniques were used to investigate the properties of the obtained elastomers, specifically tensile testing, and electrical and barrier property measurements. The deposition of IL on the GnPs’ surface positively influenced the mechanical and barrier properties of elastomers. However, samples containing graphene nanoplatelets modified from solution were characterized by less electrical conductivity. SEM analysis was additionally performed to investigate GnPs’ dispersion within SBR composites.

## 1. Introduction

Styrene–butadiene rubber (SBR) is one of the most widely used polymers in the rubber industry. The processing of polymer materials and possible application of composites, and also of SBR, are significantly dependent of fillers dispersed in the polymer matrix. In order to broaden polymers materials’ usefulness, reinforcing fillers must especially be applied. In recent years, much attention has been paid to multifunctional nanofillers, which could not only improve mechanical properties but increase electrical conductivity, as well as guaranteeing low gas permeability and the thermal stability of composites [1,2].

Graphene and other carbon allotropes have been deeply studied and their new possible applications in composites have been investigated [3,4,5,6] due to their exceptional properties [7]. Up to now, numerous researchers worked on graphene-based polymer composites [8,9,10]. However, many difficulties still need to be overcome to obtain graphene-based multifunctional composites that meet requirements. The most important limitations are: the problem of homogenous filler’s dispersion with minimal restacking within the polymer, effective mixing of graphene with polymer and the lack of understanding of interfacial interactions. Despite many studies on polymer composites containing graphene, not all such materials were characterized by simultaneous improvement in mechanical and barrier properties [11], as well as mechanical and electrical properties [12]. In some cases, the obtained results were even worse than expected due to poor graphene dispersion, the restucking of filler during the sample preparation and weak polymer–filler interfacial adhesion [13]

Many studies have been focused on improving graphene dispersion in polymer by using different methods. Among other things, it has been proposed to use ultrasonic homogenization leading to the destruction of interactions between filler particles, responsible for their aggregation [14,15]. Another solution was the use of multiwalled carbon nanotubes (MWCNTs), which due to synergistic effect had a positive effect on graphene dispersion in polymer [16,17]. Graphene modification resulting in functionalization of its surface has also been described. This was achieved using, e.g., sodium dodecyl benzene sulfonate [18], octadecylamine [15,19], silanes [12,20,21], p-phenylenediamine [22], N-1,3-dimethylbutyl-N-phenyl-p-phenylenediamine [11], maleic anhydride [23], polyvinylpyrrolidone [24], N-cyclohexyl-2-benzothiazolesulfenamide [25], natural cellulose [26], and ionic liquids [15,21,27,28,29,30,31,32].

Ionic liquids (ILs) are eco-friendly organic salts that are successfully used in many fields of polymer chemistry and technology [33]. All of this is due to their exceptional properties, e.g., extremely low vapour pressure, usually thermal stability [34]. Therefore, they have been used, e.g., to improve various nanoparticles’ dispersion in polymers [35,36,37,38,39], as a moderator of chemical reactions [40,41] or as a crosslinking substance of polymers [42]. To overcome the high activity of ionic liquids in vulcanization, influencing the safety of rubber compounds, processing supported ionic liquids-phase materials, were used. They were obtained throughout ionic liquid’s immobilization onto porous solids via various methods, e.g., impregnation, grafting, encapsulation and pore trapping [43,44]. Some studies concerned ionic liquids immobilized on the surface of fillers. Promising results were noted for carbon black modified with 1-allyl-3-methylimidazolium chloride [45,46] or carbon nanotubes modified with 1-butyl-3-methylimidazolium bis (trifluoromethylsulphonyl)imide [47] or 1-decyl-3-methylimidazolium chloride [48].

This work focuses on filling of SBR with graphene nanoplatelets (GnPs) in the presence of an ionic liquid, which was1-butyl-4-methylpyridinium tetrafluoroborate (BMPFB). Therefore, two different strategies of GnPs’ treatment with ionic liquid were employed. First, BMPFB was introduced with filler into rubber mix separately, in bulk, during its preparation on a two-roll mill, or second, BMPFB was incorporated onto the filler surface from solution. We hoped that pyridine derivative, containing unsaturated bonds, used as GnPs’ modifier would influence filler dispersion through π–π interactions between the pyridine ring and the graphenes’ surface in obtained composites. Due to that, SBR elastomers’ properties could be changed. In the present work, the relationship between the amount of proposed fillers, and the way in which the SBR/GnPs composites were prepared, was investigated using rheometric and mechanical measurements. The influence of BMPFB on filler dispersion is indirectly studied by means of thermogravimetry, electrical and barrier properties as well. Additionally, the SEM technique was applied to estimate GnPs dispersion within the polymer matrix. We expected that decorating of GnPs surface with BMPFB from solution would change the texture properties of the filler.

It is expected that these investigations can broaden knowledge about polymer processing and the application of ionic liquids in the preparation of multifunctional polymer composites.

## 2. Materials and Methods

### 2.1. Materials

Details of the materials used are listed in Table 1.

GnPs/BMPFB were prepared through the solution method. The ionic liquid was incorporated on GnPs’ surface from acetone, according to the following protocol: GnPs’ powder was dispersed easily in (CH_3_)_2_CO with the chosen IL and then was twice under sonication for 15 min, with 15 min of break. After 48 h, the resultant mixture was irradiated with ultrasound for a quarter of an hour. After 24 h, this suspension re-underwent ultrasonic treatment for 15 min. Subsequently, the resulting product was dried using a vacuum oven at 50 °C, after solvent evaporation. Thus, modified filler was used as the rubber mixes’ ingredient. The content of IL incorporated onto GnPs surface was determined using TG curves (described in the Results and Discussion section). It was necessary to develop the rubber mixes’ recipes, to ensure that all rubber compounds had equivalent GnPs concentration.

### 2.2. Preparation of SBR Vulcanizates

Experimental formulas for SBR composites’ preparation were as follows (in part per hundred parts of rubber-phr): SBR 100 phr; S 2 phr; DPG 0.5 phr; MBTS 0.5 phr; GnPs 0–5 phr; BMPFB 0–1.18 phr (for each gram of crude filler there was 1 mmole of the ionic liquid); GnPs/BMPFB 0–7.87 phr.

Rubber compounds were prepared (at temperature about 35 °C) using a laboratory two-roll mill (cylinders dimensions were: D = 150 mm and L = 300 mm) (Bridge, UK). This method was chosen to secure good processing and minimize heat buildup in rubber compound during compounds’ mixing: (i) the rolls were cooled down by cold water circulating through them, (ii) the friction between the cylinders was 1.1, (iii) the gap between rolls, time of mixing and cutting operations were adjusted. After plasticization of raw rubber, other ingredients necessary to produce rubber mix were added. Rubber sheets with a thickness of about 6–8 mm were prepared. After 48 h-seasoning at room temperature of rubber mixes, their optimal curing time was qualified using rheometric measurements. Afterwards, rubber compounds were moulded in an electrically heated hydraulic press at 160 °C to form the vulcanizates.

### 2.3. Characterization

Methods of investigations, measuring apparatus and conditions, as well as parameters obtained from these measurements, are presented in Table 2.

## 3. Results and Discussion

### 3.1. Textural Properties of Fillers

**Figure 1:**
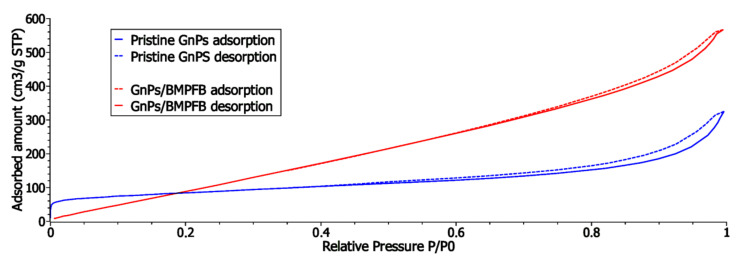
Nitrogen sorption isotherms of pristine graphene nanoplatelets (GnPs) (blue) and graphene nanoplatelets modified from solution (GnPs/BMPFB) (red) at 77 K.

**Figure 2:**
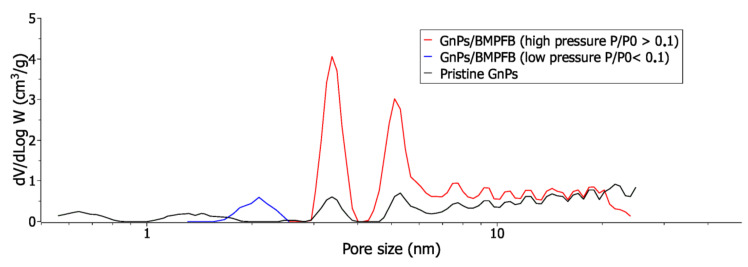
Density functional theory pore size distribution of pristine GnPs and after treatment by BMPFB.

The modification of the GnPs’ surface with BMPFB from solution was certified by measurements of the textural properties of both pristine GnPs and GnPs/BMPFB. The results are presented in Figure 1 and Figure 2 and Table 3.

Figure 1 compared the adsorption and desorption isotherms of non-modified GnPs or those modified with BMPFB from solution. An isotherm concave to the P/P0 axis adsorption at low pressure was clearly observed for pristine GnPs, which was associated with the presence of some micropores (pore size less or equal 2 nm). The isotherm, here discussed showed also a hysteresis phenomenon that indicates capillary condensation phenomena and the presence of mesopores (pore size between 2 and 50 nm). In this case, some pores exceed certain critical widths (typically Kelvin’s radius). Then, in addition to the adsorptive–adsorbent interactions, some adsorbent–adsorbent interactions appear after condensation of molecules under a multilayer form to complete the pore volumes (mesoporous volume).

In the case of the treatment of GnPs with BMPFB, the concave part of the isotherm at very low pressure is not present: a large part of filler’s microporosity would therefore be cancelled. Inversely, the treatment shows an increase in total adsorbed volume (reaching value almost two times higher than that of pristine GnPs), see Table 3. It is possible that BMPFB, which is flat around the pyridinium ring, penetrates graphene sheets that have a platelet shape, leading to some interactions between the filler and the charged ionic liquid. Thus, a huge increase in *V_MAX_* is observed. The treatment might expand the micropores to achieve the mesoporous size.

The specific surface area of the investigated fillers was determined using the Brunauer–Emmett–Teller BET model [49] and the Rouquerol curve to determine the application domain [50]. GnPs/BMPFB had a more developed surface compared to the unmodified filler (Table 3). The Dubinin–Astakhov model, which is commonly used, makes it possible to study the microporosity of the carbon [51]. In this last case, we can observe that the microporous part is more important in the pristine GnPs (this could be foreseen by the behaviour at very low pressure). The filler’s microporous surface and the microporous volume of both kinds of fillers are given in Table 3.

It was interesting to simulate the pore size distribution to know more completely the effect of the treatment. In order to achieve this, we used the DFT model [52]. For the pristine GnPs, the observed distribution was obtained by using a slit model on the carbon surface (closed porosity, As = 6) (Figure 2). In this case, we observe four different peaks: d1 = 0.6 nm; d2 = 1.4 nm; d3 = 3 nm and d4 = 5 nm (the large distribution might be attributed to the inter-particular space due to the organization of the particles).

In the case of the modified graphene, two models had to be used to simulate more neatly the isotherm: a cylindrical model at low pressure (*P/P0* < 0.1 nm) and, subsequently, a slit model (the separation of both the domains was realized according the mono–model divergence). We can note that the domains approximately separate the micropores and the mesopores. Then, we can observe that the treatment causes the micropores to disappear for the benefit of a small amount of larger cylindrical micropores (2 nm), but for greater benefit of the previous mesopores (d3 and d4) in the pristine carbon. The equivalent inter-particular spaces between both of the materials seems to show that the pores were becoming filled with molecules of modifier (BMPFB was entrapped in filler’s pores), resulting in a swelling of the pores. However, as we can observe in Figure 1, the volumes of the principal pores after treatment are largely higher than the ones of the pores in the pristine material. This could be explained by the presence of ultra-micropores (inferior to 0.8 nm) in GnPs. Some divergences can be commonly observed in the analysis of this kind of pore by N_2_ adsorption.

### 3.2. Fillers Dispersion

**Figure 3:**
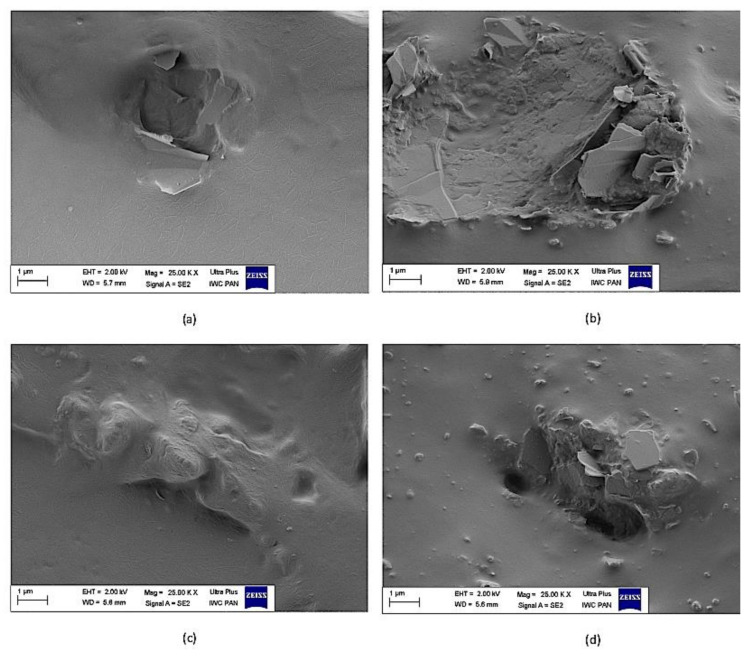
SEM micrographs of tested SBR composites filled with GnPs, modified in bulk with BMPFB: (**a**) 1phr of GnPs, (**b**) 4 phr of GnPs; and modified from solution with BMPFB: (**c**) 1phr of GnPs, (**d**) 4 phr of GnPs.

Scanning electron microscopy was useful in estimating of GnPs’ agglomeration within composites. Figure 3 shows SEM images of cross-sections of the investigated composites prepared by freeze-fracturing. Samples with 1 or 4 phr of filler were studied. Regardless of the method of GnPs’ modification with BMPFB, the tendency for GnPs to stick together and agglomerate was visible. Close-up images revealed stacking of graphene platelets. This disclosed an insufficient level of filler’s dispersion. At 4 phr of filler, some carbonous sheets were extracted from the polymer matrix, suggesting poor affinity to the polymer [53] (this is more visible for GnPs + BMPFB, see Figure 3b). This in turn can lead to a weak interphase with some voids in it [54]. However, after the GnPs’ modification from solution, SEM images registered some protuberances concealing filler’s particles in the polymer matrix. Graphene layers were wrapped by a film of SBR, thus marking a good compatibility between filler and polymer. By modification of GnPs with BMPFB from solution, the interfacial adhesion between rubber and GnPs has been improved. As the result of the above-mentioned GnPs’ treatment, no significant interfacial void formation close to the filler particles was exposed. A good compatibility of the filler particles with the polymer is crucial when obtaining polymer composite with satisfactory mechanical and barrier properties.

### 3.3. Thermal Properties of Fillers, BMPFB and SBR Composites

**Figure 4:**
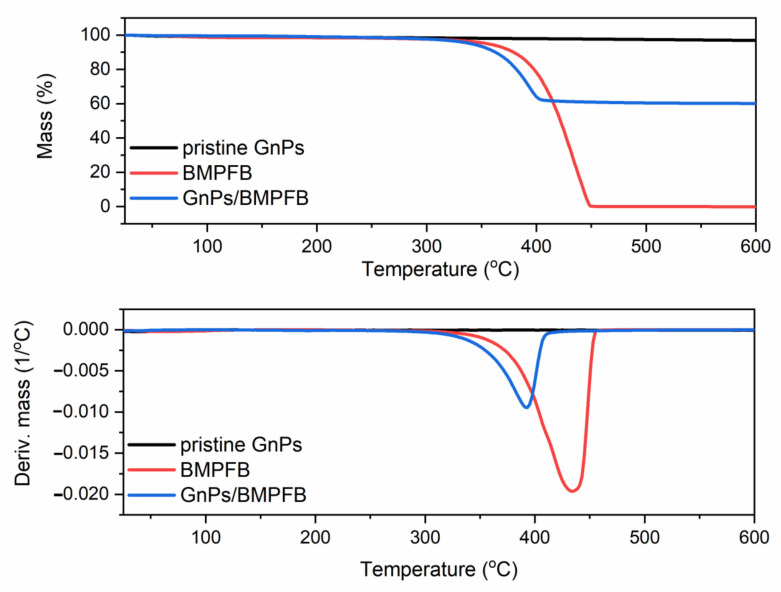
TG and DTG curves of BMPFB, pristine GnPs and GnPs modified with BMPFB from solution.

**Figure 5:**
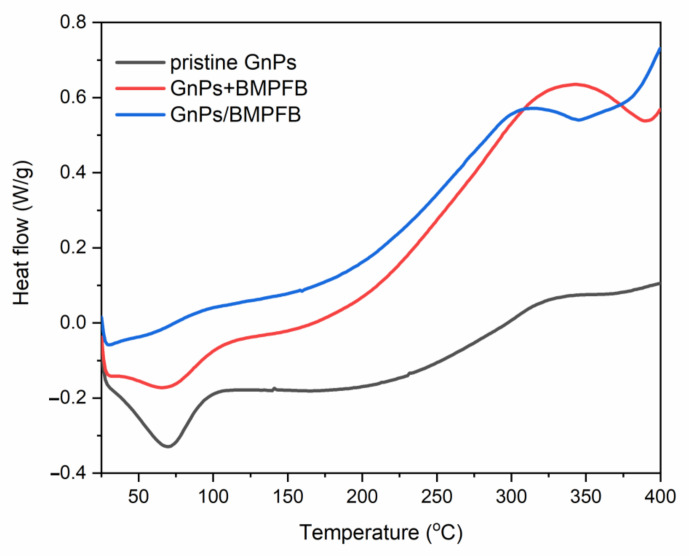
DSC thermograms of pristine GnPs, GnPs treated with BMPFB from solution or GnPs modified in bulk with ionic liquid.

Both TG and DTG analysis were useful tools for determining the thermal behaviour of BMPFB, untreated GnPs and GnPs modified with BMPFB. Some data are presented in Figure 4. In the studied temperature range, graphene was thermally stable, which was manifested by a loss of only 3% of its mass. It can be seen that thermal decomposition of BMPFB is a process with only one stage. This process is almost complete with approximately 100% mass loss. In addition, the highest BMPFB decomposition occurred at 443 °C. Since ionic liquid was settled on graphene surface, thermal stability of this filler was deteriorated by almost several dozen °C when compared to BMPFB alone. This was associated with IL degradation on the filler’s surface. It was obvious from the DTG curve that GnPs/BMPFB degradation was one-step process and was manifested as a large endothermic peak at less than 400 °C. In the temperature range of 40–100 °C, there is a loss of functional groups on the GnPs’ surface (this is also evidenced by DSC thermograms, see Figure 5). At that time, the greatest thermal effect accompanied the heating of pristine GnPs, in which functional oxygen groups were not involved in interactions with the modifier. The lowest value of the thermal effect related to the loss of functional groups from the filler’s surface was noted for GnPs/BMPFB, where the interaction of functional groups-BMPFB was the most intense, see Figure 5.

Knowing the mass loss in a sample of crude graphene, we calculated the mass loss in GnPs/BMPFB associated only with decomposition of BMPFB on the filler’s surface. When calculating the BMPFB content in the modified sample, we compared the mass loss of the sample to its total initial mass.

The effect of GnPs on thermal stability of SBR composites was also studied and data are collected in Table 4. Several papers indicated that the addition of GnPs into the polymer can significantly improve the thermal stability of the obtained material [20].

The temperature for 2% mass loss (*T*_02_) during sample decomposition was 249 °C for unfilled composite. The application of GnPs modified with BMPFB to fill SBR composites resulted in the increasing of *T*_02_ with increasing filler concentration, suggesting that incorporation of GnPs modified with ionic liquid enhanced the thermal stability of SBR. Compared to unfilled elastomer, when the GnPs’ loading was 4 phr, *T*_02_ was only 4 °C higher. It was reported that at 5 phr of GnPs, *T*_02_ equal to 237 °C was recorded. Such deterioration of thermal stability of elastomers could be due to worse filler dispersion in the polymer promoting the effect of composite thermal degradation. Taking into account that the measurement error of GnPs decorated with BMPFB led to a slight increase in the thermal stability of composites in the range of 249–257 °C, then, GnPs with surfaces that were modified with ionic liquid were a physical barrier to the SBR matrix that delayed oxygen permeation and the escape of volatile degradation products [17]. In the case of temperature for 50% mass loss during elastomer decomposition (*T*_50_), changes were not so spectacular. Generally, *T*_50_ increased in a matter that was not proportional with filler loading, when GnPs were modified with BMPFB in mass. On the other hand, as the loading of the filler decorated with BMPFB increased, *T*_50_ increased from 305 to 311 °C.

### 3.4. Cure Charcteristics

Curing characteristics including minimum torque (*M_L_*), maximum torque (*M_H_*), scorch and optimal curing time (respectively, *TC2* and *TC90*) are shown for unfilled rubber and rubber mixes filled with GnPs in Table 5. *M_L_* is a measure of uncured compounds’ viscosity and *M_H_* is a measure of the stock modulus of the cured compounds [55]. The introduction of GnPs into rubber mixes caused an increase in *M_L_* and *M_H_* with filler loading when compared with the control sample, i.e., unfilled. These changes were more pronounced when GnPs were decorated with BMPFB. It is known that an efficient elastomer curing is fundamental to produce composites with satisfactory properties. Some authors noticed that the presence of carbon filler led to *TC2* shortening, probably due to an increment in thermal conductivity in presence of those fillers that promoted fulfilment of curing [56]. Our research did not confirm this. Conversely, the addition of modified graphene and its amount did not significantly affect *TC2*, considering measurement discrepancy. These observations are in line with previous studies regarding carbon-based fillers and their role in delaying the onset of vulcanization [57]. The basic accelerators’ additives are absorbed on the filler’s surface, which also led to an increase in *TC90*. This occurs owing to the active centers on the filler’s surface, which favor the absorption of curing system compounds, e.g., accelerators of curing. Such behaviour was noticed for carbon fillers [58]. Generally, while an increase in *TC2* may be a desirable phenomenon that increases the safety of blends’ processing, an increase in *TC90* is disadvantageous from a technological point of view. Note that when filler concentration increased to 4 and 5 phr, *TC90* shortened, including standard errors (in the case of rubber mixes with GnPs and BMPFB introduced separately into SBR). Such an effect could be assigned to the filler’s ability to moderate the vulcanization reaction. Previously, it was postulated that GnPs accelerate the reactions between curing system compounds during active complex formation [35]. Presumably, as GnPs’ loading is higher, some part of the filler absorbed curing accelerators and the rest participated in interactions with curing system compounds. Moreover, the presence of GnPs (however modified with BMPFB) caused an increase in torque increment during vulcanization (Δ*M* being the difference between *M_H_* and *M_L_,* and the measure of crosslink density of elastomers [59]) in relation to unfilled sample. It is probable that the interaction in the filler–polymer interphase was then higher.

Applying DSC analysis (a commonly used technique to study the curing kinetics of polymers [40]), the impact of GnPs modification with BMPFB on the temperature and energetic effects of vulcanization was investigated.

**Figure 6:**
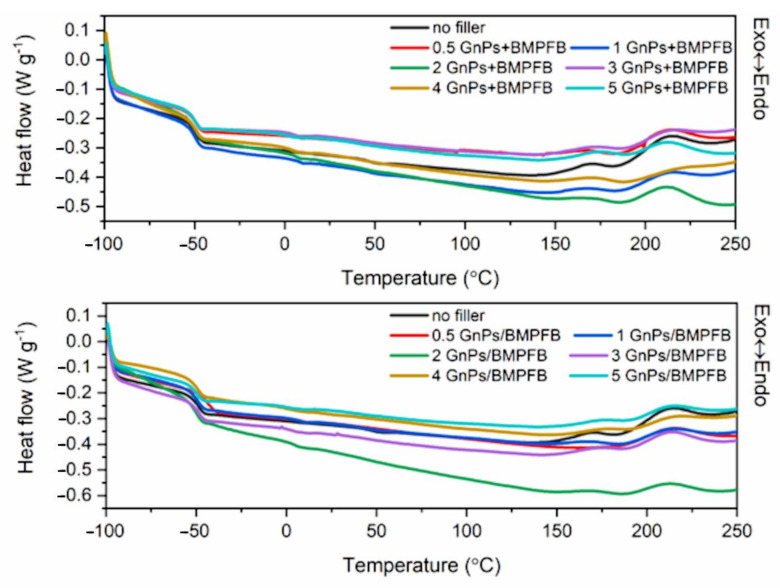
Heat flow response of elastomers filled with GnPs modified with BMPFB in bulk or from solution.

The results for filled compounds and reference sample are shown in Figure 6 and are summarized in Table 5, accounting for standard errors. From the presented data, it appeared that vulcanization of unfilled rubber mix occurred in a range of 154–230 °C. The energetic effect was 10.1 Jg^−1^. It should be remembered that lowering the onset vulcanization temperature is beneficial for technological reasons because it allows rubber to be crosslinked at lower temperature. When GnPs were modified with BMPFB through the melt mixing method, the *T_C_* and Δ*H_C_* of those samples showed no systematic change with filler loading. It is worth noting that among the samples discussed, the lower the amount of GnPs, the lower the onset vulcanization temperature was. When a compound contained only 0.5 phr of filler, this parameter was equal to 144 °C (for SBR/GnPs + BMPFB compound) and 146 °C (for SBR/GnPs/BMPFB compound). However, 5 phr of GnPs in compounds led to an increase in onset vulcanization temperature, with it reaching 159 °C and 158 °C (respectively, for SBR/GnPs + BMPFB and SBR/GnPs/BMPFB). The endset vulcanization temperature was higher by a few °C compared to the value of this parameter characterizing the sample without filler. The exception was a sample with 2 phr GnPs. In the case of samples filled with GnPs decorated with BMPFB, it was noticeable that the onset and endset vulcanization temperature, respectively, were raised and lowered with the increase in filler loading. In turn, Δ*H_C_* decreased with higher amounts of GnPs, on which BMPFB was deposited. It is obvious that using different GnPs modified with BMPFB led to no significant changes in the *T_g_* values of elastomers in comparison to the unfilled sample (Table 5). These results indicated that the incorporation of GnPs modified with BMPFB did not affect the average relaxation of bulk segments [54]. These results did not demonstrate any tendency regarding filler loading or the methods of its modification. Therefore, from the technological point of view, it can be said that there are no differences in the temperature range in which these elastomers can be used.

### 3.5. Mechanical Performance

The tensile modulus at 100% elongation (*M100*), tensile strength (*TS*) and elongation at break (*EB*) of the investigated elastomers are shown in Table 6. The mechanical strengthening of polymer composites can be achieved, e.g., by filler’s functionalization [15]. It is likely that filling the composites with GnPs in the presence of BMPFB would have an effect on the mechanical properties of the investigated samples since IL containing an aromatic part in its structure was treated as a coagent of sulfur curing. Hence, the pyridine rings from IL form hard domains within the polymer structure after curing [60]. If so, BMPFB could participate in the transferring of stress via “filler” particles or act as a stress-redistributor when polymer chains scission occurs. Among the tested samples, the lowest *M100* value with regard to standard deviation was recorded for unfilled composite. Higher *M100* values were exhibited by composites containing GnPs modified from solution.

Modification of GnPs with BMPFB from solution resulted in the enhancing of the filler’s roughness and the increasing of its surface area and thus generated, on the filler’s surface, a higher number of contact points with the polymer. In order to ensure effective load transfer from GnPs to the polymer, such treatment was advantageous. BMPFB used to modify GnPs hindered their stacking in polymer to a certain extent (see Figure 3), leading to better filler dispersion. The prominent reinforcement effect of GnPs could be attributed to their better dispersion and stronger filler–polymer and filler–filler interactions. Therefore, better filler dispersion led to effective stress transfer at the interfacial region and enhanced mechanical properties of samples. Similar conclusions were drawn in the case of carbon nanotubes [61]. At the highest concentration of GnPs modified with BMPFB from solution, *TS* was more than 100% higher when compared with sample containing the lowest loading of such filler, and was 2.5-fold higher when compared to unfilled sample. Taking into account the measurements’ discrepancy, no significant differences in *TS* were achieved for vulcanizates containing from 2 to 4 phr of GnPs, regardless of how it was modified. Usually, loading composites with fillers leads to a decrease in *EB* [59]. For the studied filled samples, the opposite effect was observed. It was noted that graphitic fillers were susceptible to disturbing the physical crosslinking sites of styrenic-based elastomers, thus facilitating polymer chain movement [62]. Herein, the increase in the elongation at break could be explained by the plasticizing effect of the neat GnPs, enabling the polymer chain movement. Even if development of rubber composites with a combination of high strength and higher extensibility simultaneously is a challenge, the results show that SBR composites containing GnPs modified with BMPFB (through two different methods) simultaneously had improved *TS* and *EB*. Such an effect may be a sign of additional crosslinks’ formation. Owing to this, the mechanical stress acting on the filler was better dispersed in the polymer, and filler particles pulling out from the polymer matrix were reduced. This was in agreement with SEM images.

Shore A hardness (*H*) of all composites are provided in Table 6. As expected, the filled composites had higher *H* when compared with unfilled sample (taking into account standard error). The more uniform the GnPs’ dispersion within the polymer, the harder the composites [12]. Thus, regarding the measurement error, *H* values that were slightly higher when GnPs were decorated with BMPFB can be explained by stronger interfacial interactions and better filler dispersion.

### 3.6. Electrical Propertiesof SBR Composites

**Figure 7:**
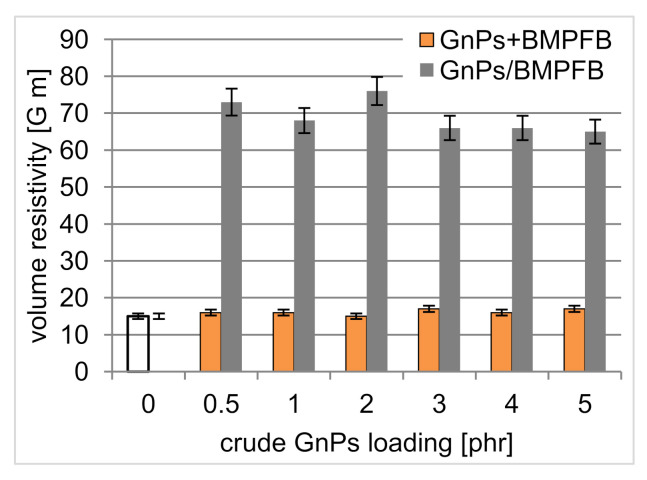
Dependence of SBR composites volume resistivity (*ρ*) on pristine GnPs loading.

Several factors, e.g., concentration as well as filler dispersion within polymer have crucial meaning for the enhancement of electrical conductivity of polymer composites [63]. One of the ways to increase the electrical conductivity of elastomers is to introduce conductive fillers, for example GnPs [56]. Figure 7 shows the electric volume resistivity (*ρ*) of SBR composites fabricated with the use of GnPs modified during rubber mix preparation on two-roll mill or from solution. Additionally, reference sample was investigated. Thereby, GnPs’ loading in investigated composites changed from 0 to 5 phr (and was equal to 0, 0.23, 0.45, 0.91, 1.36, 1.82 and 2.27 vol.%, respectively). Surprisingly, adding GnPs and BMPFB separately to rubber mix did not affect the electrical conductivity of composites. Despite the presence of ionic liquid and filler with exceptional electron mobility, the *ρ* of these composites remained practically unchanged compared to unfilled composite. Usually, at higher concentrations of conducting filler and with finer dispersion, the inter-filler distance becomes smaller, which enables the formation of paths for electron mobility [8]. It is worth noting that applying GnPs decorated with BMPFB did not give rise to a significant increase in electrical conductivity. On the contrary, despite better dispersion of these GnPs within SBR, the formation of conduction pathways was difficult. Otherwise, their presence would guarantee an increase in electrical conductivity. It is likely that the mobility of ions of BMPFB, which was immobilized on the surface of GnPs, was then significantly reduced. This way BMPFB deposited on GnPs surface was not active in the electric conductivity process and additionally blocked GnPs, thus preventing the movement of electrons. This is supported by *ρ* values that were higher than those of composites containing GnPs and BMPFB introduced into rubber mixes separately. *ρ* was reduced by only a few units with increasing filler loading in SBR/GnP + BMPFB composites. Certainly, even at the high GnPs concentrations used in the investigated SBR vulcanizates, the percolation threshold was not achieved.

### 3.7. Gas Barrier Properties

In this study, the gas barrier properties of SBR composites filled with GnPs as a function of filler loading and the method of GnPs’ modification were investigated. The gas permeability coefficient (*P*) and gas transfer rate (*GTR*) are collected in Table 7.

In the case of unfilled composite, *P* was 2.57 mole m^−2^ s^−1^ Pa^−1^ and *GTR* was 2.1 × 10^−4^ m^2^ s^−1^ Pa^−1^. Taking into account standard errors, the addition of GnPs and BMPFB into composites led to *P* and *GTR* increments with increasing filler loading from 0.5 phr to 3 phr, reaching a value of 4.93 mole m^−2^ s^−1^ Pa^−1^ and 4.0 × 10^−4^ m^2^ s^−1^ Pa^−1^ (respectively, for *P* and *GTR*). Further increasing of GnPs concentration caused a decrease in *P* and *GTR*. This may indicate a deterioration of GnPs’ dispersion in composites. At higher GnPs loading, despite the worse dispersion, filler acts as a gas barrier, which extends the diffusion path through polymer. It was discovered previously that fine dispersion of graphene sheets within polymer prolonged tortuous paths for gas diffusion [64]. It appeared to be the case that GnPs decorated with BMPFB had a beneficial effect on the barrier properties of composites. This could indicate better filler dispersion within the polymer matrix.

## 4. Conclusions

Graphene nanoplatelets modified with 1-butyl-4-methylpyridinium tetrafluoroborate were prepared using two different strategies of modification. The first strategy involved introducing ionic liquid with filler during the rubber mix preparation (melt-mixing method), and the second strategy involved the incorporation of modifiers onto the filler’s surface from solution. This strategy of modification led to significant changes in the textural properties of thus obtained filler in comparison with untreated graphene nanoplatelets. As the specific surface area of modified filler developed its dispersion in the polymer was enhenced thus influencing on some composites’ properties. This new strategy of filler modification with ionic liquid was advantageous for load transfer effectiveness. Styrene–butadiene rubber composites containing modified GnPs improved *TS* and *EB* simultaneously. Among the two kinds of fillers used, graphene nanoplatelets decorated with ionic liquid had a beneficial effect on barrier properties and led to an enhancement in the thermal stability of composites. Despite its better dispersion in elastomers, no increase in elastomers’ electrical conductivity was observed. It is likely that this filler limited the formation of conduction pathways in composites.

## Figures and Tables

**Scheme 1:**
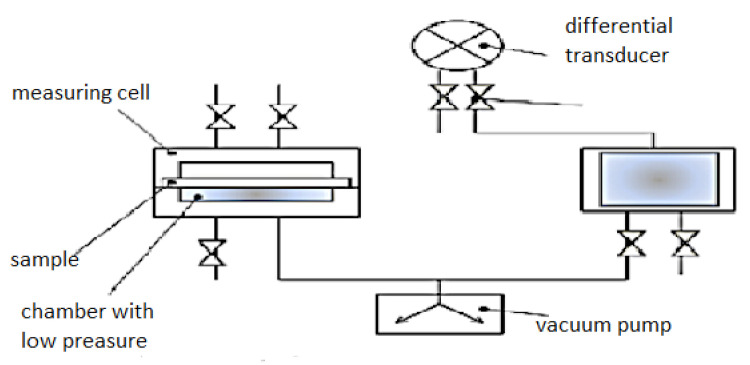
Diagram of the apparatus for gas permeability measurements of studied styrene–butadiene rubber composites.

**Table 1:** Characteristics of the materials used.

Material	Symbol of Material	Supplier	Characteristics
Styrene–butadiene rubber (KER 1500)	SBR	Synthos S.A., Oswiecim, Poland	23% of styrene mers
1,3-diphenylguanidine	DPG	Akrochem Co., Akron, OH, USA	m.p. 143 °C
2,2′-dibenzothiazyl disulfide	MBTS	Akrochem Co., Akron, OH, USA	m.p. 172 °C
Sulfur	S	Siarkopol, Tarnobrzeg, Poland	industrial grade
Graphene nanoplatelets powder	GnPs	XG Sciences Inc., Lansing, MI, USA	high purity, *S.A.* = 300 m^2^ g^−1^
1-butyl-4-methylpyridinium tetrafluoroborate	BMPFB	IoLiTec GmbH, Heilbronn, Germany	purity 99%
Graphene nanoplatelets decorated with ionic liquid	GnPs/BMPFB	Synthesized by our own	
Acetone	(CH_3_)_2_CO	POCh, Gliwice, Poland	purity 99.5%

m.p. melting point, *S.A.* surface area.

**Table 2:** Methods of samples’ characterization.

Method of Characterization	Apparatus	Conditions	Parameters
Rheometric measurements	a rotational rotorless rheometer D-RPA 3000 (MonTech, Buchen, Germany)	const. temperature (160 °C) const. frequency of oscillation (1.7° 0.1 Hz) const. amplitude of oscillation (3°)	M_L_, M_H_, TC90, TC2
Tensile testing	Zwick, model 1435, Ulm, Germany	crosshead speed 500 mm min^−1^, room temperature	M100, EB, TS
Hardness testing	type A durometer (Zwick Roell, Ulm, Germany)	apparatus force 10 N	H
Thermal properties	TGA/DSC instrument (Mettler Toledo, Greifensee, Switzerland)	calibration with In and Zn; samples’ heating from 25 to 600 °C in an Ar, next in an air from 600 °C to 900 °C (20 °C min^−1^), steady gas flow of 50 mL min^−1^	T_02_, T_50_, Δm
Thermal properties	DSC analyzer (Mettler Toledo, Greifensee, Switzerland)	samples’ heating from 25 to 600 °C (10 °C min^−1^) in Ar	BMPFB content in GnPs/BMPFB
Curing kinetics testing	DSC analyzer (Mettler Toledo, Greifensee, Switzerland)	calibration with In and n-octane; samples’ cooling to −100 °C (10 °C min^−1^), then heating up to 250 °C (10 °C min^−1^) under nitrogen purging	T_g_, T_C_, Δc_pC_
Electrical properties	MIC-100 resistance meter (Sonel, Swidnica, Poland)	type EP-1 of measuring electrodes	ρ
Gas permeability	home-made apparatus (see Scheme 1)	air, room temperature; low pressure (less than 10 Pa) with a vacuum pump	GTR, P
SEM	LEO 1450 (Carl Zeiss AG, Oberkochen, Germany)	carbon film sputtering	filler’s dispersion
Texture properties	ASAP 2420 (MICROMETRICS, Norcross, GA, USA)	degassing: on degas port 12 h/25 °C; on analysis port 2 h/25 °C; low pressure dosing 10 cm^3^/g	V_MAX_, S_BET_, S_DA_, V_DA_

**Table 3:** The textural properties of pristine graphene nanoplatelets (GnPs) and GnPs modified with BMPFB from solution (GnPs/BMPFB).

Type of Filler	Parameter				
	*V_MAX_*, cm^3^ g^−1^ STP	*S_BET_*, m^2^ g^−1^	*S_DA_*, m^2^ g^−1^	*V_DA_*, cm^3^ g^−1^	Pore Size Distribution
Pristine GnPs	325	298 ± 1	325	0.115	multi-modal
GnPs/BMPFB	567	636 ± 10	203	0.072	bimodal

*V_MAX_*—maximal adsorbed volume, *S_BET_*—specific surface area, *S_DA_*—microporous surface, *V_DA_*—microporous volume.

**Table 4:** TG data of SBR composites.

Vulcanizate	*T*_02_**/°C	*T*_50_/°C
No filler	249	301
05GnPs + BMPFB	243	297
1GnPs + BMPFB	247	299
2GnPs + BMPFB	251	307
3GnPs + BMPFB	251	305
4GnPs + BMPFB	253	305
5GnPs + BMPFB	237	295
05GnPs/BMPFB	249	305
1GnPs/BMPFB	251	307
2GnPs/BMPFB	257	309
3GnPs/BMPFB	255	309
4GnPs/BMPFB	253	311
5GnPs/BMPFB	255	311

*T*_02_—initial decomposition temperature of sample, *T*_50_—temperature for 50% mass loss during sample decomposition (standard deviation of *T*_02_ and *T*_50_ ± 1 °C).

**Table 5:** Properties of SBR compounds.

Rubber Compound	*M_L_*/dNm	*M_H_*/dNm	*TC2*/min	*TC90*/min	*T_C_*/°C	Δ*H_C_*/Jg^−1^	*T_g_*/°C
No filler	0.57	7.22	2.0	16.8	154–230	10.1	−49.4
05GnPs + BMPFB	0.61	7.32	2.2	19.6	144–235	9.9	−49.5
1GnPs + BMPFB	0.64	7.44	2.4	20.6	149–232	5.9	−50.3
2GnPs + BMPFB	0.67	7.56	2.4	21.1	152–215	9.9	−49.5
3GnPs + BMPFB	0.71	7.65	2.3	21.2	149–234	6.3	−50.0
4GnPs + BMPFB	0.73	7.67	2.2	19.8	151–238	3.5	−50.0
5GnPs + BMPFB	0.76	7.87	2.0	19.1	156–234	7.9	−50.1
05GnPs + BMPFB	0.61	7.37	2.3	19.5	146–248	11.7	−49.3
1GnPs + BMPFB	0.63	7.25	2.6	21.1	151–234	6.5	−49.8
2GnPs + BMPFB	0.70	8.31	2.4	20.2	153–233	6.0	−50.8
3GnPs + BMPFB	0.74	7.86	2.5	21.4	154–235	9.0	−49.2
4GnPs + BMPFB	0.80	8.29	2.5	21.4	158–237	4.9	−50.3
5GnPs + BMPFB	0.75	8.04	2.6	21.7	158–234	6.1	−50.0

*M_L_* minimum torque, *M_H_* maximum torque, *TC2* scorch time, *TC90* optimal curing time, *T_C_* vulcanization temperature range, Δ*H_C_* enthalpy of vulcanization, *T_g_* glass transition temperature (standard deviations of *M_L_* ± 0.02 dNm, *M_H_* ± 0.1 dNm, *TC2* ± 0.1 min, *TC90* ± 0.4 min, *T_C_* ± 1 °C, Δ*H_C_* ± 0.7 Jg^−1^ and *T_g_* ± 0.1 °C).

**Table 6:** Mechanical properties of SBR vulcanizates.

Vulcanizate	*M100*/MPa	*EB*/%	*TS*/MPa	*H*/°Sh A
No filler	0.89 ± 0.03	320 ± 8	2.16 ± 0.10	32 ± 1
05GnPs + BMPFB	0.90 ± 0.01	386 ± 5	2.29 ± 0.13	35 ± 1
1GnPs + BMPFB	0.92 ± 0.02	400 ± 8	2.84 ± 0.12	36 ± 1
2GnPs + BMPFB	1.01 ± 0.02	440 ± 12	3.78 ± 0.16	36 ± 1
3GnPs + BMPFB	0.96 ± 0.01	473 ± 6	3.96 ± 0.12	35 ± 1
4GnPs + BMPFB	0.96 ± 0.02	480 ± 13	3.82 ± 0.06	36 ± 1
5GnPs + BMPFB	1.04 ± 0.01	476 ± 12	4.71 ± 0.18	36 ± 1
05GnPs/BMPFB	0.90 ± 0.01	354 ± 10	2.75 ± 0.13	33 ± 1
1GnPs/BMPFB	1.02 ± 0.02	391 ± 8	4.18 ± 0.16	36 ± 1
2GnPs/BMPFB	1.11 ± 0.02	414 ± 8	5.31 ± 0.12	36 ± 1
3GnPs/BMPFB	1.14 ± 0.01	427 ± 8	5.32 ± 0.11	38 ± 1
4GnPs/BMPFB	1.22 ± 0.01	438 ± 7	5.40 ± 0.11	38 ± 1
5GnPs/BMPFB	1.31 ± 0.01	414 ± 7	5.86 ± 0.29	38 ± 1

*M100*—modulus at 100% relative elongation, *EB*—elongation at break, *TS*—tensile strength, *H*—hardness.

**Table 7:** Gas barrier properties of SBR vulcanizates.

Vulcanizate	*GTR*/10^−4^ mole m^−2^ s^−1^ Pa^−1^	*P*/mole m^−1^ s^−1^ Pa^−1^
No filler	2.1	2.57
05GnPs + BMPFB	1.5	1.86
1GnPs + BMPFB	1.8	2.21
2GnPs + BMPFB	3.9	4.88
3GnPs + BMPFB	4.0	4.93
4GnPs + BMPFB	3.4	4.25
5GnPs + BMPFB	2.8	3.51
05GnPs/BMPFB	1.7	2.09
1GnPs/BMPFB	1.8	2.21
2GnPs/BMPFB	3.3	2.80
3GnPs/BMPFB	1.5	1.81
4GnPs/BMPFB	1.5	1.87
5GnPs/BMPFB	2.1	2.56

*GTR*—gas transfer rate, *P*—gas permeability coefficient (standard deviation of *GTR* ± 0.30 × 10^−4^ mole m^−2^ s^−1^ Pa^−1^ and *P* ± 0.31 mole m^−1^ s^−1^ Pa^−1^).
